# Evaluating a Preventive Heart Health Program for Women at Midlife: Protocol for a Mixed Methods Pilot Study

**DOI:** 10.2196/83574

**Published:** 2026-05-25

**Authors:** Grace Shu Hui Chiang, Cheryl Pei Ting Tan, Christine Mei Leng Tioh, Eunice Wei Xin Lee, Laureen Yi-Ting Wang

**Affiliations:** 1 Department of Medicine, Alexandra Hospital, 378 Alexandra RoadSingapore, 159964, Singapore, +65 69082222; 2 Department of Cardiology, National University Heart Centre, National University Health System, 5 Lower Kent Ridge Road, Singapore, Singapore; 3 Yong Loo Lin School of Medicine, National University of Singapore, Singapore

**Keywords:** preventive health, health coaching, behavior change, lifestyle modifications, cardiovascular

## Abstract

**Background:**

Cardiovascular disease (CVD) is the leading cause of death in women. Risk factors can be compounded by hormonal changes, especially during the menopause transition. Positive health promotion through a behavioral change strategy may be the most effective approach to reducing CVD mortality and morbidity.

**Objective:**

Health care providers are instrumental in identifying and managing CVD risk factors in women. This study aims to explore and assess the barriers and facilitators to implementing preventive heart health care in Singapore.

**Methods:**

A prospective mixed methods pilot study using both quantitative and qualitative research methods for program implementation and evaluation through an interrupted time series study design will be conducted with a sample of 100 women aged 45 to 65 years. An A-B-A reversal design of 6-month intervals (baseline [A]—participants’ healthy heart behaviors before the introduction of health coaching, intervention [B]—health coaching is introduced and participants’ behaviors are measured to observe for changes from baseline, and return to baseline [A]—health coaching is removed and there is a return to baseline) will be used to assess the feasibility and acceptability of behavioral change strategies to increase heart-healthy habits. Data collection will occur over 3 phases: the preimplementation phase, the implementation phase, and the postimplementation phase. Quantitative data collection will include structured surveys. Qualitative interviews will be conducted among a subset of patients and health care providers for the exploratory evaluation of this study. Purposive sampling will be used for the recruitment of interview participants. Primary outcomes will be the reach, effectiveness, adoption, and feasibility of the preventive heart health program. Secondary outcomes will include laboratory blood test results and CVD risk assessment. A segmented regression analysis of interrupted time series will be used to evaluate the impact of the behavioral change strategy intervention. Qualitative data will be analyzed using an inductive approach, and thematic synthesis will be used to identify patterns. Integrating quantitative and qualitative data will facilitate a nuanced understanding of the barriers and facilitators of implementing a preventive heart health program among midlife women.

**Results:**

This study was funded in May 2022. Data collection started in April 2024 and is projected to end in April 2026. A total of 60 participants had been enrolled as of December 2025. Data analysis is expected to commence in June 2026 and expected results published in the autumn of 2026.

**Conclusions:**

Experiential knowledge arising from the adoption of personalized health coaching for midlife women can help ascertain the contextual challenges and effectiveness of service implementation in Singapore. Study findings can contribute to refining the framework of sex-specific, tailored CVD prevention care; enable upscaling of such services to other hospitals; and improve future preventive care for midlife women.

## Introduction

Cardiovascular disease (CVD) is a global public health crisis accounting for 32% of all deaths globally, and it is projected that 23.6 million people will die from CVD by 2030 [1]. CVD accounted for approximately 30% of all deaths in Singapore in 2021 [2] and was the leading cause of disease burden in 2010, responsible for approximately 20% of total disease and injury burden [3].

Although women tend to develop CVD 10 years later in life than men, the health outcomes for women, including mortality and prognosis, are often poorer [4-6]. CVD is the leading cause of death in women [1,7], and 1 in 3 women dies from heart-related conditions [8]. In Singapore, CVD in women causes more morbidity and mortality than breast and cervical cancers [9]. This disease burden is expected to increase as Singapore’s population ages and more women outlive men. Primary and secondary prevention strategies, such as therapeutic lifestyle changes in tandem with a multidrug regimen targeting major CVD risk factors, are established measures to lower the burden of CVD [10,11].

Women with risk factors such as diabetes or hypertension have a higher average lifetime risk for heart attacks than men with similar risk factors [12]. The incidence of fatal coronary heart disease (CHD) is higher in older women [13], while improvements in CHD morbidity and mortality in younger women (<55 years) have not increased [14,15]. Clinical studies have found that women with CHD have a higher burden of cardiovascular risk factors than men [14,16]. Moreover, there are women-specific CVD risks, such as previous pregnancy complications, breast cancer, or premature menopause [17]. While men have a 2-fold higher incidence of CHD and related mortality than women, with increasing age, this gap in mortality and morbidity shrinks as older women experience greater incidences of heart disease [13].

Unique conditions such as takotsubo cardiomyopathy (also called transient apical ballooning and stress cardiomyopathy) and microvascular ischemia disproportionately affect menopausal women [18]. However, CVDs in women are underdiagnosed and undertreated [19]. Women frequently underestimate their CVD risks, and physicians often do not identify CHD as a prominent cause of morbidity and mortality in women, leading to delays in diagnosis and treatment [12,20,21]. Women are often labeled anxious, or symptoms are attributed to menopause [18]. Women are less likely to receive statins or optimal medical therapy [18]. In Singapore, there is an alarming decline in awareness of the severity of CVD. In the recent 2020 Singapore Heart Foundation Women’s Heart Health Survey, only 9% of respondents were aware that CVD was the leading cause of death among women in Singapore, a decrease from the already low proportion of 10% in 2016 [22].

A woman’s risk for heart disease accelerates during the menopause transition [12,23]. During this transition, women experience disruptive changes to both their reproductive organs and physical and psychological well-being. Management issues range from short-term symptoms, such as insomnia and palpitations, to intermediate issues, such as bone health, or longer-term issues related to developing CVD. Endocrine and metabolic changes associated with menopausal transition, such as increased abdominal fat accumulation, increased insulin resistance, dyslipidemia, and endothelial dysfunction, suggest a predisposition to an increased CVD risk [23]. Early management of the traditional risk factors of CVD is essential, and the American Heart Association has guidelines for multidisciplinary care to prevent CVD in women [17,24]. Other countries, such as Canada, have dedicated groups to tackle this problem [21,25]. Collaborative care involving cardiologists, gynecologists, and women’s heart centers has been established as essential for preventive care in women [26]. In addition, strategies focusing on CVD risk reduction in younger women are essential to reduce the high cardiovascular burden among South Asian women.

Motivational interviewing strategies in health coaching can help patients achieve optimal cardiovascular health by inculcating various healthy habits and supporting lifestyle changes, including diet, physical activity, nicotine avoidance, sleep, weight maintenance, blood lipid control, glucose control, and blood pressure control [24]. Moreover, a personalized and culturally appropriate approach would be beneficial in supporting augmented cardiovascular preventive efforts in the years preceding menopause [24], leading to an improvement in health outcomes and quality of life (QOL) in women undergoing menopause. Research on health coaching for women undergoing menopause has been promising in improving health outcomes [27], but such preventive care has not been extensively evaluated as a clinical service in Singaporean women. Hence, there is a need to assess the viability of implementing a preventive women’s heart health program and investigate the barriers and facilitators associated with implementing such a service in Singapore.

Both the Proctor framework [28] and RE-AIM (reach, effectiveness, adoption, implementation, and maintenance) [29] have been recommended for use to evaluate effective implementation of evidence-based interventions in CVD [30]. As it might be challenging to distinguish between health coaching (behavioral intervention) and the implementation strategy, the preventive women’s heart health program adopted the Proctor framework [28] (ie, acceptability, adoption, appropriateness, cost, feasibility, fidelity, penetration, and sustainability), which would allow for deeper analysis of optimal strategies to promote evidence-based program implementation (health coaching to improve healthy heart behaviors) and the approach to executing health coaching in midlife women as the implementation strategy. The RE-AIM framework [29] was used to explore external validity and evaluate implementation strategy success.

This study could illustrate how health coaching can be developed as an additional clinical support strategy to enhance positive health promotion in Singapore. There is a need for programs of behavioral modification to be trialed in a controlled fashion and assessed rigorously for efficacy. Using health services research and implementation science will be crucial in driving this strategy. Implementation science and conceptual frameworks will provide a crucial lens to better understand CVD prevention programs in women at midlife and define successful strategies that enhance the delivery, sustainment, and effects of CVD prevention programs in Singapore.

Promising strategies on behavior modification can be tested using multicenter implementation trials. The implementation of this program in Singapore has wider implications due to the ethnic differences in disease burden. Contextualizing the customary practices and lifestyle factors, including diet, exercise, smoking, and psychosocial stress, is important. Cultural attitudes toward treatment adherence may also be important and can influence patient health outcomes.

This protocol paper describes a prospective mixed methods pilot study to assess the viability and effectiveness of implementing a preventive women’s heart health program using a behavioral change strategy to increase heart-healthy habits among women.

The prospective mixed methods study aims to address the following:

- Identify barriers and facilitators associated with sustaining a preventive heart health program among women.
  - Hypothesis: the preventive women’s heart health program could improve awareness of CVD and encourage adoption of positive heart health behaviors and habits.
- Identify microlevel, mesolevel, and macrolevel factors associated with the provision of a women’s preventive heart health program.
  - Hypothesis: receipt of preventive heart health will be associated with microlevel factors (eg, daily procedures and interactions between patients and health care providers), mesolevel factors (eg, local health service, community elements, such as patient and health care provider attitudes and support), and macrolevel factors (eg, health education policies, health care accessibility, and health care affordability).
- Understand health care provider and patient perceptions of the provision and use of preventive heart health care among women.
  - Hypothesis: patterns of preventive heart health care use and delivery are influenced by patient and health care provider perceptions and experiences.

## Methods

### Design

This study uses a mixed methods design augmenting quantitative analyses with qualitative assessments of patients and health care providers through an interrupted time series (ITS) study design, a robust quasi-experimental design that is often used to evaluate real-world feasibility and effectiveness of behavioral change interventions [31]. A mixed methods study was adopted to provide greater insight, deeper understanding, and more comprehensive analysis of the connections and contradictions between quantitative and qualitative data. The quantitative component provides a numeric description of participants’ attitudes and behaviors, while the qualitative component provides context and explores their subjective perceptions and experiences. The pilot study will be conducted over a period of 12 months.

### Systematic Review

We conducted a systematic review of women’s heart health policies to enhance understanding of current recommendations and gaps at the macrolevel [32]. This review was used to consolidate current research findings and policies to identify gaps in women’s heart health practice. The review screened 21,476 records and synthesized results from 124 English-language publications worldwide. Using a life course approach, we assessed the connection between clinical recommendations and policy and documented global recommendations and policies addressing prevention of CVD in women. Specifically for midlife and menopausal women, we found that an interdisciplinary approach adopting both lifestyle changes and pharmacological interventions should be introduced in perimenopausal (transitional period before menopause) women to minimize cardiovascular risk [32].

### Patient Population and Selection

This pilot study is conducted in a Women’s Heart Health Clinic in a restructured public hospital in Singapore. A total of 100 English-speaking participants aged 45 to 65 years who attend the Women’s Heart Health Clinic will be invited to participate in this study. Participants will be approached either while waiting to see their health care provider in the clinic or after the clinic visit. Women who are pregnant, outside the menopausal transition age range of 45 to 65 years, or unable to provide informed consent will be excluded from the study.

For the semistructured survey and interview, purposive sampling (ie, the deliberate choice of a participant due to particular qualities they possess) will be used to recruit at least 5 women aged between 45 and 65 years who have attended the preventive heart health program and 10 health care providers aged 21 years and above who are involved in this program. There will be a specific focus on recruiting patients representative of a broad range of CVD risk factors and health literacy levels. The concept of information power will be used to guide final sample size for the qualitative interviews based on the following considerations: (1) whether the study aim is broad or narrow, (2) dense or sparse sample specificity, (3) application or nonapplication of an established theory; (4) quality of the dialogue, and (5) whether case or cross-case analysis, with cross-case requiring more participants. Information power indicates that the more information the sample holds relevant to the actual study, the lower the number of participants required [33].

### Data Collection

Quantitative data regarding participants’ clinical status, symptoms, and self-administered surveys will be collected 6 months before the intervention, during the 6-month intervention period, and 6 months after the intervention to capture level and trend changes of one or more outcomes over time. Upon recruitment and preintervention assessment at the baseline visit, participants will undergo personalized health coaching. Compliance will be tracked through health logging and follow-up assessments. Participants will also undergo interviews and surveys to evaluate changes in physical activity, diet scores, and QOL.

Qualitative interviews will be conducted with these participants and health care providers using a one-to-one semistructured questionnaire carried out either in person, over the telephone, or via video call to obtain feedback and explanatory data about the program.

### Conceptual Framework

The proposed research will use various implementation science frameworks to answer questions regarding future scale-up and long-term sustainability of the intervention if it is determined to be effective. These conceptual frameworks will aid in evaluating how individual and contextual variables influence program implementation and acceptability.

**Figure 1.**
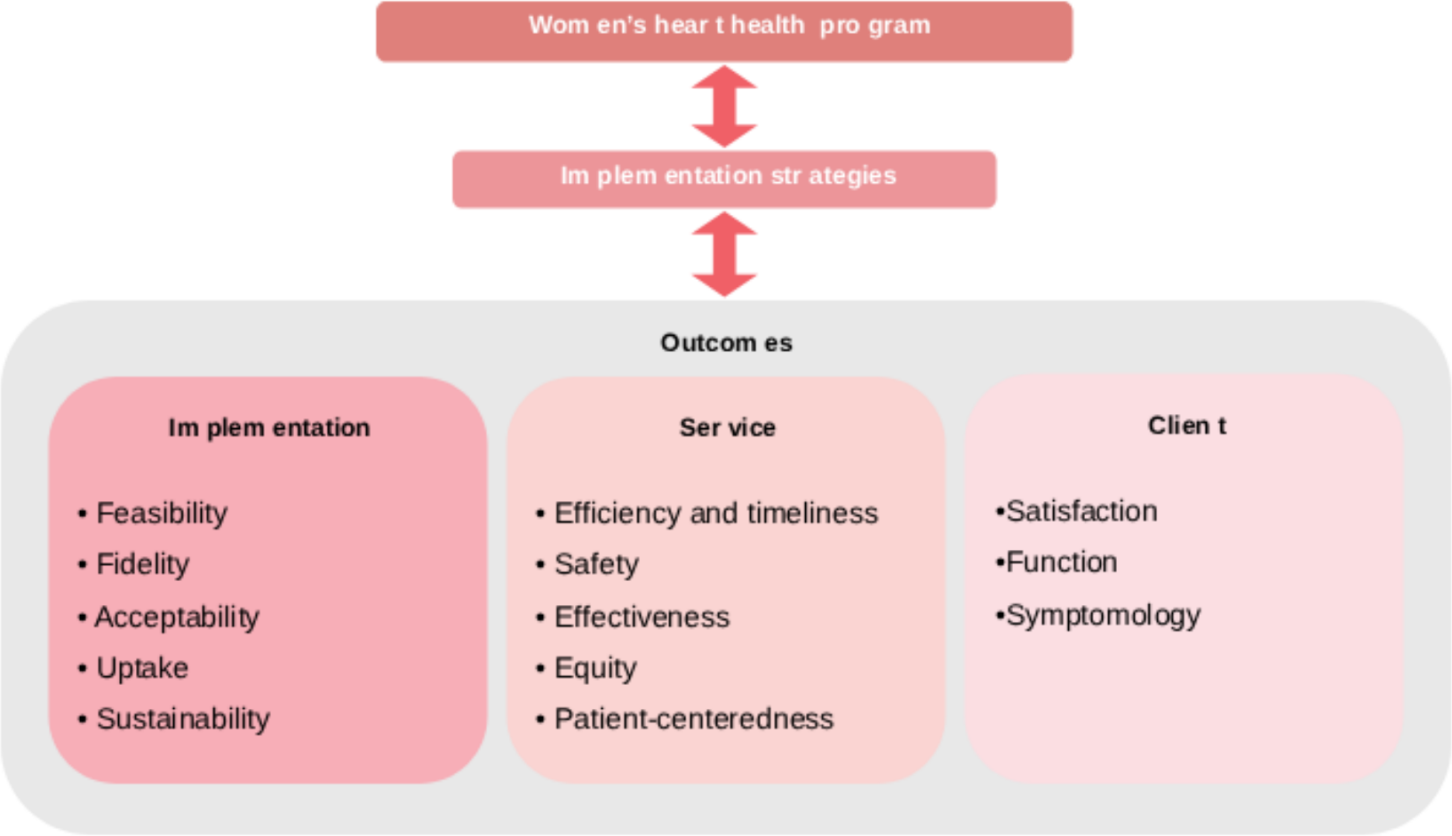
Framework adapted from Proctor conceptual model of implementation research [28].

We adopted the Proctor framework (Figure 1 [28]) to ensure the inclusion of essential implementation science outcomes and measurements with respect to users, stakeholders, and the context of the intervention, which were then measured using a mixed methods approach involving validated quantitative scales and in-depth interviews where applicable.

The in-depth questionnaires were constructed from both the Theoretical Domains Framework (TDF) [34] and capability, opportunity, motivation, and behavior (COM-B) models [35] to understand the underlying individual and contextual determinants that can positively impact programs implementation using behavior change strategies and how individuals adopt and maintain health behavior changes.

In addition, we used the RE-AIM framework [29] to guide the planning and evaluation of the program to identify the contextual barriers and facilitators associated with implementation success. This involves gaining insight from previous evidence and key stakeholders in health care at the macrolevels, mesolevels, and microlevels (illustrated in Multimedia Appendix 1) to understand how multiple interventions can be effectively packaged, integrated, and delivered within outpatient clinic settings.

Investigating at the microlevel would allow us to relate to the day-to-day practices and interactions between patients and health care providers. Investigating the mesolevel would allow us to understand local health service and community factors, such as attitudes and support from patients and health care providers involved in the women’s heart health program. Exploring the macrolevel would help identify the interrelated network among all core stakeholders within the health care system that are essential to the success of this program. Findings elucidated at the respective levels will then be used to iteratively improve the current pilot program strategies and address identified barriers where possible.

### Intervention Design

The women’s preventive heart health program is a multidisciplinary program that aims to prevent the onset of CVD by empowering women with the knowledge and skills to achieve optimal cardiovascular health even after they have exited the program.

**Figure 2.**
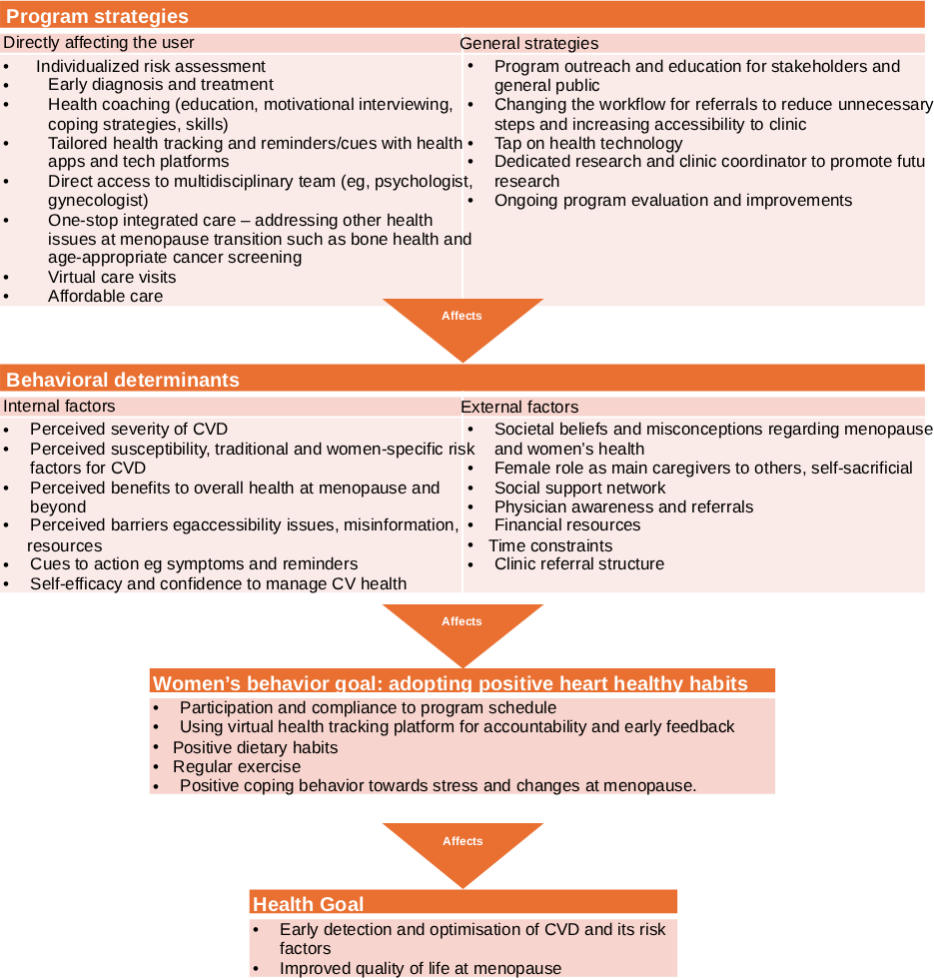
An integrated behavioral determinant intervention logic model for cardiovascular disease (CVD) screening and prevention in midlife women.

The integrated behavioral determinant intervention logic model for CVD screening and prevention in midlife women was used to devise the program and is elaborated upon in Figure 2. The logic model of the problem was established following the PRECEDE model [36], which puts the diagnosis into action and measures the program's effectiveness. A logic model of change (program strategies) was then developed to describe what needs to change in behavior at the individual and environmental levels. Logic models have been used to design interventions for cardiac populations in previous studies [37,38].

For instance, women underestimating their CVD risks is an internal barrier, whereas underdiagnosis and undertreatment of CVD in women by physicians are external barriers [12,20,21]. Program strategies to tackle these barriers would include individualized risk assessment and sex-specific tailored care for women undergoing the menopause transition, and increased physician awareness.

The intervention comprises women having access to trained onsite personalized health coaching according to their CVD risk levels, health goals, and lifestyle. Health coaching sessions will be conducted by certified health coaches within the hospital sites on a one-to-one basis in the Women’s Heart Health Clinic. Collaborative consultations between the physician and health coach will include motivational interviewing to identify any barriers causing the knowledge and execution gap and target changes in both internal and external factors of behavioral determinants, ultimately helping participants attain health goals. Information provided to the participants by the physicians and health coaches is aligned with the national clinical practice, dietary, and physical activity guidelines and personalized where appropriate.

Follow-up visits with the physician and health coach will be aligned to the A-B-A reversal design of 6-month intervals (baseline [A]—participants’ healthy heart behaviors before health coaching (intervention) is introduced will be measured to establish a pretreatment level, intervention [B]—biweekly health coaching is introduced and participants’ healthy heart behaviors are measured to see if it changes from the baseline level, and return to baseline [A]—health coaching is removed and there is a return to baseline conditions, participants’ healthy heart behaviors are monitored to determine whether their behavior reverts to baseline, thus confirming that the intervention has caused the change), concluding with the postintervention time point. Clinical data collection and questionnaires will be administered during those visits. Relevant outcomes will be collected during the follow-up visits.

Women will be encouraged to track their health parameters using the existing hospital eHealth platform between the follow-up visits. Timely encouragements and reminders relevant to individuals’ targets set together with the health coach will also be sent via the eHealth platform.

Women will be stratified into low-, moderate-, or high-risk groups based on whether their individualized health goals are achieved, symptoms are relieved, and program participation is completed. Low-risk women will be discharged to their family physician with detailed handovers and health targets, while moderate- to high-risk women may continue in the program.

**Figure 3.**
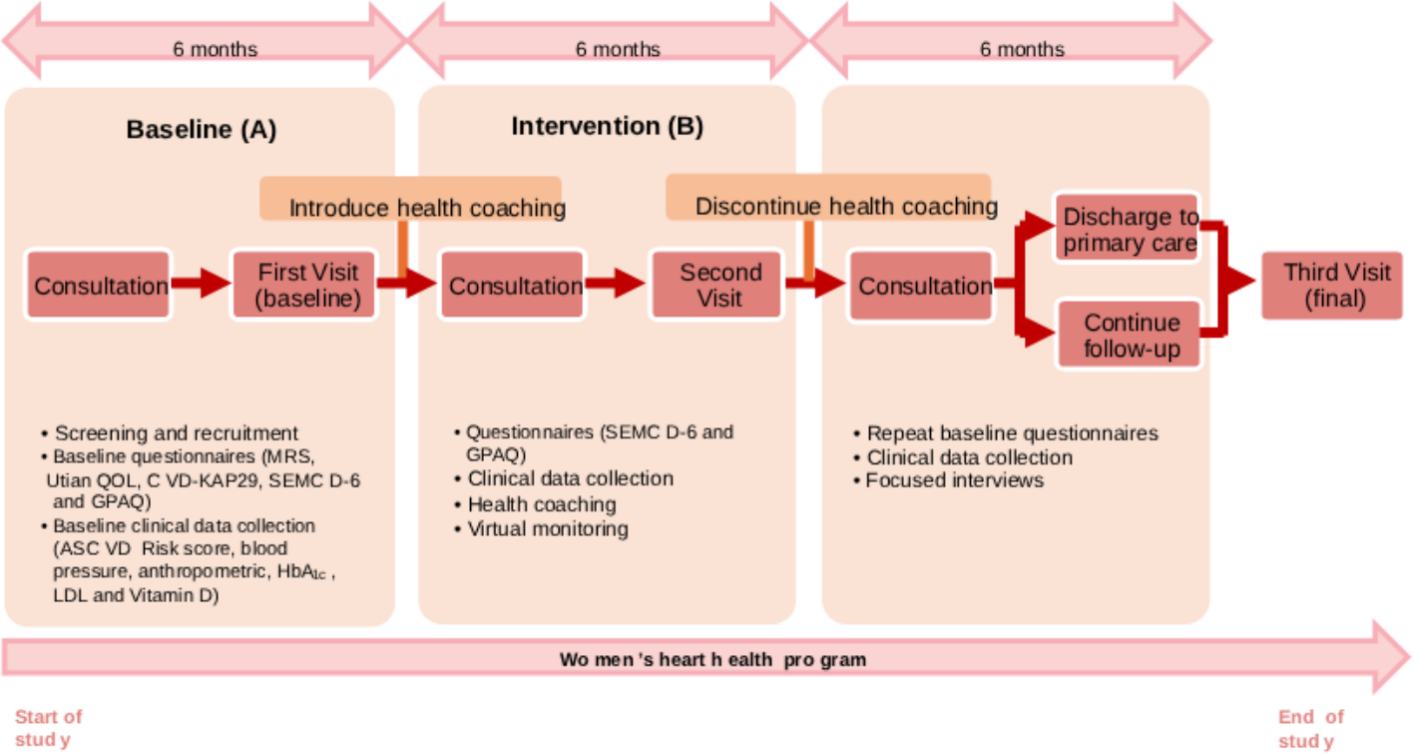
Summary of the preventive heart health program study design. ASCVD: atherosclerotic cardiovascular disease; CVD-KAP29: cardiovascular disease knowledge, attitude, and practice questionnaire; GPAQ: Global Physical Activity Questionnaire; HbA_1c_: hemoglobin A_1c_; LDL: low-density lipoprotein; MRS: Menopause Rating Scale; QOL: quality of life; SEMCD-6: Self-Efficacy to Manage Chronic Disease 6-Item Scale.

One-to-one semistructured interview sessions with participants and health care providers lasting approximately 30 to 45 minutes each will be conducted at the end of the study. The interviews will be conducted during the last visit or at a time and place convenient for the participant. The TIDieR (Template for Intervention Description and Replication) checklist [39], including the description of the behavior change intervention is presented in Checklist 1. A summary of the program is shown in Figure 3.

### Outcome Measures

#### Primary Outcomes

We used the RE-AIM model [29] to conceptualize the primary outcomes because it is well suited to the practical approach of this study and provides a comprehensive framework for assessing complex interventions in real-world practice beyond controlled trial conditions. It also allows for translation of the intervention between individual and institutional levels to promote uptake, transferability, and eventual public health impact of the program.

Reach indicators involve assessing the number, proportion, and representativeness of the women participating in the program. Effectiveness is defined as the degree to which participants engage in healthy heart behaviors and the degree of change in clinical CVD indicators and QOL measures. Both behavioral change and patient-related outcome indicators will be measured. Adoption of program strategies by the health care providers in the Women’s Heart Health Clinic will be reviewed from the perspective of physicians, allied health professionals, and the clinical operations team. The following implementation strategies will be used and assessed: evaluation and feedback, training and supporting stakeholders, adaptation and tailoring, infrastructure change, and financial strategies. To assess the continuation and maintenance of the program strategies and practices after the study completion, the following aspects will be considered: feasibility, sustainability, service outcomes, and participant satisfaction in the form of observations, audits, and feedback (refer to Multimedia Appendix 2 for further details of the 5 stages).

#### Secondary Outcomes

Sociodemographics, anthropometric measurements (height, body weight, and BMI), blood pressure measurements, laboratory blood test results (hemoglobin A_1C_, lipid panel, and vitamin D levels), and CVD risk assessment documentation will be collected from the clinic records at all 3 visits.

### Questionnaires

Questionnaire responses relating to lifestyle (Global Physical Activity Questionnaire [GPAQ] and Self-Efficacy for Managing Chronic Disease 6-Item Scale [SEMCD-6]) will be self-administered by participants at all 3 time points. Questionnaires pertaining to patients’ knowledge of CVD (CVD-knowledge, attitude, and practice [KAP29]), menopausal symptoms (Menopause Rating Scale), and QOL (Utian QOL Scale) will be self-administered at both baseline and final visits.

Physical activity behavior is assessed using the GPAQ [40], a tool that evaluates the moderate-to-vigorous physical activity time and sedentary time during a week. Physical activity has been associated with reduced CVD incidence and mortality [41]. The GPAQ has been used to evaluate physical activity behavior in menopausal women in previous studies [42].

Self-efficacy and patient engagement will be assessed using the SEMCD-6 questionnaire [43]. The SEMCD-6 has been applied to evaluate the effect of self-management programs in CVDs [44,45].

The KAP regarding CVD, CVD risk factors, and CVD symptoms will be evaluated using the CVD-KAP29 questionnaire [46].

Menopausal symptoms will be assessed using the Menopause Rating Scale [47], a validated tool that is widely used to assess symptom severity in midlife women [48]. It has been used in a previous study conducted among midlife Singaporean women [49].

The Utian QOL Scale [50] will be used to quantify the sense of well-being in midlife women. It is a validated tool that has been used in Asian populations [51,52].

Routine clinic business and operations data obtained from the clinic operations team and physical or cardiovascular lab results available during the study that are not collected as part of the research procedures will also be reviewed. Recordings of the relevant health parameters will also be collected from an online health monitoring platform. All data will be deidentified and kept confidential.

Semistructured interview guides have been developed for patients and health care providers (refer to MultimediaAppendices 3  4). The semistructured interviews were developed based on the constructs of the TDF [34] and COM-B models [35] and refined by the research team. The TDF can be used as a lens to explore adherence to heart-healthy habits and provides comprehensive insight into key influences on the targeted behavior which are affective, cognitive, social, and environmental in nature [53]. The TDF integrates 33 theories of behavior and behavior change into 14 domains which have been shown to be related to healthy heart behaviors [53]. The COM-B model [35] was applied alongside the TDF to provide a comprehensive, theory-driven evaluation of participants’ beliefs and motivations regarding adherence to healthy heart behavior. Previous studies adopting the COM-B model have been effective in understanding complex health behaviors [54]. For participants, we will investigate feasibility, fidelity, acceptability, uptake, sustainability, and patient-centeredness. For health care providers, we will investigate knowledge, skills, roles, beliefs, perceptions, barriers, and facilitators.

### Statistical Analysis

#### Quantitative Data

Data from the structured surveys will be analyzed quantitatively using appropriate statistical software. Exploratory analyses will be performed, including testing assumptions of linearity, homoscedasticity, multicollinearity, and normality of errors and, if necessary, transformation of data. Baseline characteristics, preimplementation and postimplementation survey findings, and CVD indicators will be reported descriptively using univariate methods such as chi-square tests and 2-tailed *t* tests. Comparisons between preprogram and postprogram outcomes will be made using paired *t* tests and Wilcoxon rank-sum test. Complete case analysis will be used as the primary analysis. As the main focus of this pilot research is to establish the implementation of a new intervention, the study is not powered to detect between-group differences. Variables will thus be provided descriptively as well as using paired *t* tests where appropriate. As this is a program evaluation of a pilot program with a small sample size, statistical manipulations, such as multiple imputations will not be performed.

A segmented regression analysis of ITS will be used to evaluate the impact of the behavioral change strategy intervention. Data collected over a 1-year period will be plotted. The clearly defined intervention period and the availability of at least 10 data points before, and 10 data points after the intervention enabled this quasi-experimental design. The analysis can estimate the intervention effect while accounting for time trend and autocorrelation among the observations. The ITS design allows the estimation of any sudden change in level immediately after the intervention, defined as the difference between the observed level at the first intervention time point and that predicted by the preintervention time trend, estimation of the difference between preintervention and postintervention slopes, and estimation of the level effect 6 months after the intervention. The level effect at 6 months after implementation is defined as the difference between the predicted value at 6 months after the intervention calculated using the preslope and the observed value at 6 months after implementation. After testing the absence of first-order autocorrelations with the Durbin-Watson statistic, a time series regression model, an autoregressive integrated moving average time series regression model without adjustment for autocorrelation will be fitted to the data. Analyses will be performed using SPSS statistical software (IBM Corp).

#### Qualitative Data

Interviews will be transcribed verbatim. Data from the qualitative interviews will be managed using NVivo (Lumivero), a qualitative data analysis software package. Two members of the research team will independently code the data and assess the information power of the sample. Codes will be assigned to text segments that represent distinct ideas or concepts and subsequently grouped into broader themes through iterative discussion. These 2 individuals, together with the study team, will work collaboratively to interpret the findings.

Data analysis will follow Clarke and Braun reflexive thematic analysis approach [55], which is theoretically independent and can be used for a wide range of research questions, data collection methods, and sampling methods. Themes are conceptualized as patterns of shared meaning across the dataset based on an underlying central concept or idea that provides an answer to the research question: “Evaluating a preventive heart health program for women at midlife.” Themes will be generated predominantly inductively from the data. However, some predetermined concepts will guide the first level of analysis. These are based on the literature, the study objectives, and from lived experience. The predetermined concepts are reflected in the interview questions. Data analysis will follow a rigorous process including several steps: (1) data familiarization, (2) coding, (3) generating initial themes, (4) reviewing themes, (5) defining and naming themes, and (6) writing up. Coding will occur at 2 levels—semantic and latent—with the objective of identifying themes across the entire dataset.

For the integrative merging of qualitative and quantitative data, a description of whether each variable (ie, laboratory blood test results, CVD risk assessment, physical activity, and menopausal symptoms) postintervention assessment was better, worse, or the same as at baseline will be used. These variables will be imported into NVivo using a Microsoft Excel spreadsheet. A series of interactive cross-tabulation matrices will be generated using the NVivo query function, for example, to compare perspectives regarding physical activity improvements among participants whose GPAQ scores improved and those whose GPAQ scores worsened at the postintervention assessment. This process will contribute to the systematic development of in-depth qualitative themes, thereby supporting the development of meta-inferences, or conclusions drawn from the merged qualitative and quantitative datasets, and avoiding simplistic quantification of qualitative data (ie, counting coded references) as a means of data integration [56].

### Ethical Considerations

This study has been approved by the National Healthcare Group Domain Specific Review Board (2022/00350) on August 15, 2023. Any modification to the study protocol would require preapproval from the committee. Written informed consent will be obtained from all participants involved in the study. To ensure participant privacy, all data will be anonymized before analysis. Personal identifiers such as names and contact details will be removed from all transcripts and datasets. Each participant will be assigned a unique identifier, and only anonymized data will be used in the analysis. Audio and visual recordings will be securely stored and destroyed after transcription. Access to the data will be restricted to the research team, and all sensitive data will be stored on encrypted hospital servers to ensure confidentiality. Participants will be reimbursed SG $20 (US $15.60) for each study visit.

## Results

This study was funded in May 2022. Data collection started in April 2024 and is projected to end in April 2026. A total of 60 participants had been enrolled as of December 2025. Data analysis is expected to commence in June 2026 and expected results published in October of 2026.

## Discussion

### Anticipated Findings

This study plans to design, develop, test, and evaluate a preventive heart health program for midlife women. We adopted various implementation science frameworks, such as Proctor framework [28] and RE-AIM [29], to provide “real-world” insights into how a sex-specific, tailored CVD program may benefit midlife women.

Previous studies indicate that although women can recognize common CVD risk factors, they often fail to apply this knowledge to themselves. As a result, even women with multiple risk factors may not view themselves as being at risk, and this low perceived personal susceptibility can reduce engagement in preventive behaviors [57]. Moreover, major lifestyle changes can be difficult to adopt or sustain, particularly when health messages are delivered through a top-down approach. However, research indicates that women are more likely to relate to heart health strategies when they are presented in ways that feel personally meaningful [58]. To address this, we developed a preventive heart health program that comprises a personalized health coaching intervention tailored according to participants’ CVD risk levels, health goals, and lifestyle.

### Strengths and Limitations

Because of the nature and context of the preventive heart health program, the use of an ITS approach is the most appropriate to estimate effects of behavioral change strategy as randomization is not feasible. This approach can provide valuable insights into the effectiveness of the program by assessing the changes over time and is more sensitive to differences in intervention effects, as it involves self-comparison of the participants, which controls for confounding variables to the greatest extent possible. It can also be conducted with a small sample size, which is ideal for our pilot study. The application of a mixed methods approach also allows for a comprehensive and nuanced understanding of the barriers and potential feasibility issues associated with this program.

Despite the benefits of an ITS approach, the lack of randomization is a limitation that restricts the ability to draw definitive conclusions regarding the effects of the program. Another limitation of the study protocol is the reliance on self-administered questionnaires for subjective measurements, which may induce response bias and limit the depth of responses. The study team has mitigated this limitation by including semistructured interviews, which can bridge these gaps by allowing the team to probe deeper into responses, clarify ambiguities, and address complex topics. This approach will result in more detailed and nuanced data.

### Future Directions

The intended result of this study is to evaluate the effectiveness and feasibility of a preventive heart health program among midlife women. By recognizing barriers and facilitators associated with the sustainability of a preventive heart health program in midlife women, identifying microlevel, mesolevel, and macrolevel factors associated with the provision of such a program, and enhancing understanding of health care provider and patient perceptions of preventive heart health care in women, this study will enable us to further refine the process and framework of sex-specific, tailored CVD prevention care, thereby enabling the upscaling of such services to other hospitals and improving future preventive care for midlife women.

## Supplementary material

10.2196/83574 Multimedia Appendix 1 Levels of the health care system.

10.2196/83574 Multimedia Appendix 2 Outcome measurements based on Proctor and RE-AIM framework.

10.2196/83574 Multimedia Appendix 3 Semistructured interview guides.

10.2196/83574 Multimedia Appendix 4 Feedback form for program participants.

10.2196/83574 Checklist 1 TIDieR checklist.

## Abbreviations

CHD: coronary heart disease
COM-B: capability, opportunity, motivation, and behavior
CVD: cardiovascular disease
GPAQ: Global Physical Activity Questionnaire
ITS: interrupted time series
KAP: knowledge, attitude, and practice
QOL: quality of life
RE-AIM: reach, effectiveness, adoption, implementation, and maintenance
SEMCD-6: Self-Efficacy for Managing Chronic Disease 6-Item Scale
TDF: Theoretical Domains Framework
TIDieR: Template for Intervention Description and Replication
