# Supplementary figures and images for: Evaluating a Preventive Heart Health Program for Women at Midlife: Protocol for a Mixed Methods Pilot Study
Source: JMIR Res Protoc. 2026 May 25;15:e83574. doi: 10.2196/83574 (PMC13200805; doi:10.2196/83574)

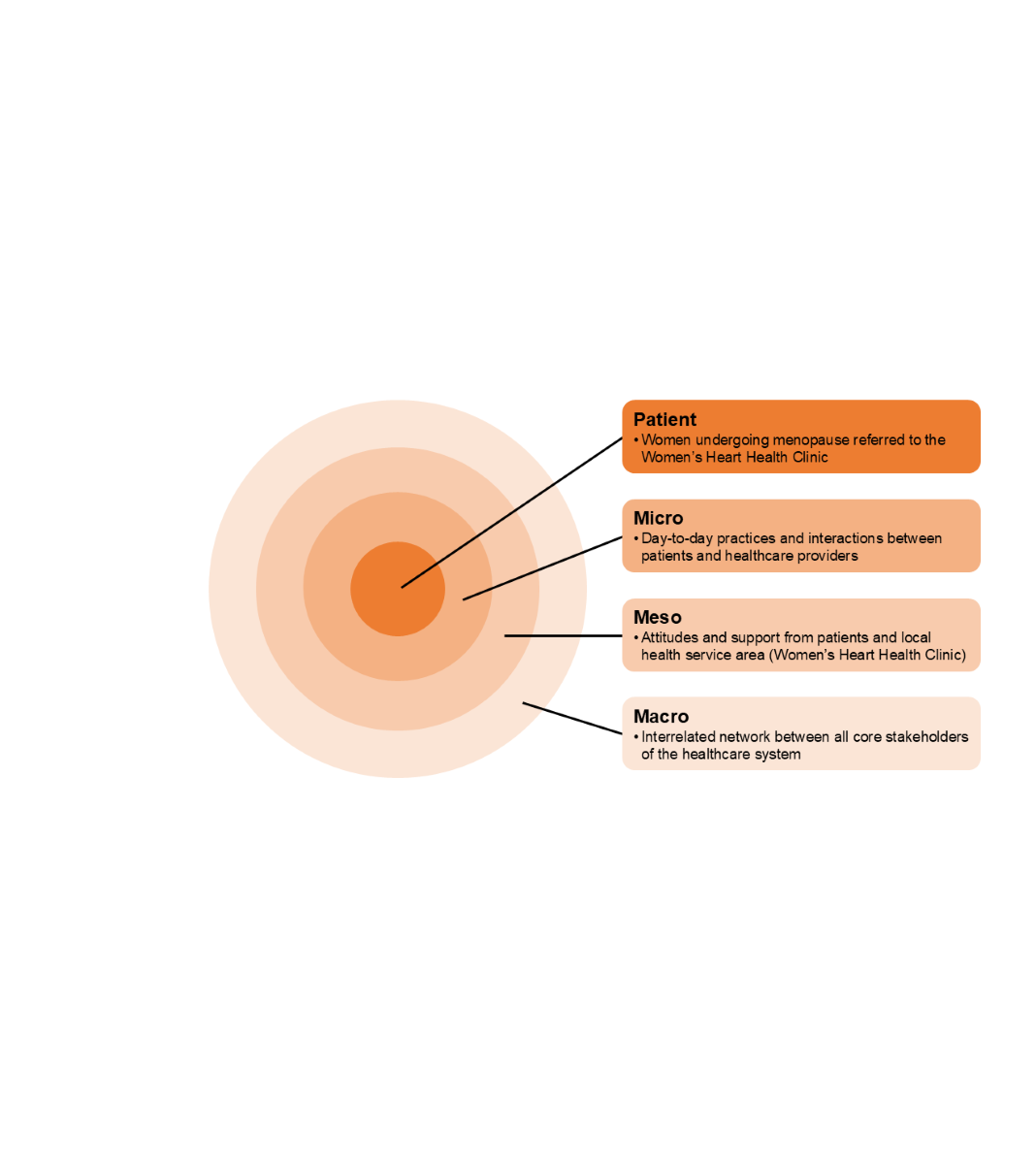

Supplement: Multimedia Appendix 1 [file resprot-v15-e83574-s001.png]
