# Supplementary material for: Evaluating a Preventive Heart Health Program for Women at Midlife: Protocol for a Mixed Methods Pilot Study
Source: JMIR Res Protoc. 2026 May 25;15:e83574. doi: 10.2196/83574 (PMC13200805; doi:10.2196/83574)
Supplement: Multimedia Appendix 2 [file resprot-v15-e83574-s002.docx]

| **Implementation Outcomes** | | |
| --- | --- | --- |
| **Programme Evaluation**  **Questions** | **Measurements** | **Method** |
| **Feasibility**   - Are the resources (manpower, funds, equipment, knowledge) to carry out the intervention sufficient? - Is the intervention suitable   for the intended population? | - The proportion of women who complete the programme - Feedback survey - Qualitative interviews with programme providers and stakeholders | Surveys, interviews, observation/direct counts |
| **Fidelity**  - How is it determined if the intervention was successfully delivered? | - Healthcare provider: Use of menopause transition health screening tool checklist by physicians - Participants: Compliance to   health logs of blood pressure and glucose | Observations, checklists, self- reporting |
| **Acceptability**  - What are providers’ & participants knowledge, attitudes and experience  regarding the intervention? | - Feedback forms - Qualitative interviews | Surveys, interviews |
| **Uptake**   - How can we know if employees utilised the intervention? - Who is the target audience,   and where are they being targeted? | - Referral numbers and participation rate | Direct counts, administrative data |
| **Sustainability**   - Can the hospital maintain the initiative for the long- term? - How likely is it that this program will have lasting effects on individuals? - How can information regarding the programmes progress and evaluations be   disseminated? | - Biannual audits post study - Sharing sessions at clinical board meetings | Audit of records, interviews |

| **Service Outcomes** | | |
| --- | --- | --- |
| **Programme Evaluation**  **Questions** | **Measurements** | **Method** |
| **Efficiency and timeliness**  - What is the rate of  improvement? | - Time to completion of programme | Direct counts, data analysis |

| - Are the processes meeting the timeline targets? | - Rate of improvement in various health score aspects in patients clinical and behavioural  outcomes |  |
| --- | --- | --- |
| **Safety**  - Are there any safety issues? | - Audits and feedback | Observations, audits, surveys |
| **Effectiveness**  - What is the efficacy of this  programme? | - Improvement in clinical and behavioural measures | Biomarkers, questionnaires |
| **Equity**  - Is there equality in the outreach and delivery of the  service? | - Referral numbers and participation rate | Direct counts and observations |
| **Patient centeredness**  - Do individuals feels that the programme is participant-  centric? | - Feedback and qualitative interviews | Surveys and interviews |

| **Client/Patient-related Outcomes** | | |
| --- | --- | --- |
| **Programme Evaluation**  **Questions** | **Measurements** | **Method** |
| **Physical activity**  - How do we tell that the participants have behaviour change regarding physical  activity? | - Global Physical Activity Questionnaire (GPAQ) [40] | Questionnaire |
| **Specific to women at midlife QOL scale**  - How is it determined if quality of life and symptoms relating to peri-menopause  have improved? | - Utian Scale (encompasses occupational, emotional, sexual, health QOL aspects) [50] | Questionnaire |
| **Menopause symptoms**  - How is it determined if quality of life and symptoms relating to peri-menopause  have improved? | - Menopause rating scale [48] | Questionnaire |
| **Self-efficacy**  - How do we know participants are able to continue on measures on their own? | - Self-efficacy for managing chronic diseases 6-item scale (SEMCD- 6) [43] | Questionnaire |
| **Knowledge, attitudes, practices (KAP) score**  - Does the programme result in changes of KAP in participants? | - Modified CVD-KAP29 [46] | Questionnaire |

| **Future risk of CVD**  - Does the programme help to lower CVD risk? | - Score calculated by healthcare professional - Pooled cohort equation to predict   10-year risk for CVD | Calculation of validated risk score |
| --- | --- | --- |
| **Clinical measurements**  - Does the programme result in improvements in clinical indicators? | - BP, Weight and BMI - Blood biomarkers:   - Lipids   - HbA1c   - Vitamin D | Anthropometric measures  Laboratory tests |
| **Participant satisfaction**  - Are participants satisfied  with programme? | - Feedback forms (Appendix 5) | Questionnaire |
