# Supplementary material for: Evaluating a Preventive Heart Health Program for Women at Midlife: Protocol for a Mixed Methods Pilot Study
Source: JMIR Res Protoc. 2026 May 25;15:e83574. doi: 10.2196/83574 (PMC13200805; doi:10.2196/83574)
Supplement: Multimedia Appendix 3 [file resprot-v15-e83574-s003.docx]

**Semi-structured interview guide for patients**

***Behaviour changes, believes and overall health***

1. How are you feeling right now?
2. How do you feel about your overall health & wellbeing now?
   - Using a percentage, can you give your health and wellbeing a score before and after the clinic follow-up.
3. Do you now feel more in control/confident about managing your heart health and wellbeing from menopause and beyond?
4. How is your exercise frequency?
5. - This includes taking conscientious action to decrease sedentary time, such as, choosing to take the stairs instead of the elevator.
6. What do you do to increase your daily physical activity levels?
   -Give some examples.
7. How is your diet compared to before these sessions?
   -how often do you eat fast food/what is in a typical meal)
8. How is your sleep and mood?
9. Do you have any difficulties with taking your medications daily ?
10. What are the proactive steps you have tried to incorporate for a healthier lifestyle?
11. Do you set yourself any health goals?
12. Do you think you can sustain these efforts after being discharged from the clinic?
13. What do you think will happen if you stop these efforts?
14. Do you try to give lifestyle advice to your family and friends regarding their heart health?
    -Prompts: examples, such as preparing healthier meals for your family members, asking them to go for walks with you, etc.

***Patient perceptions of feasibility, acceptance, feedback of programme***

1. What are your thoughts on the women’s heart health preventive programme?
2. For yourself and for other women at mid-life - Why do you think women visit the doctor? Is it mainly for acute illnesses or insurance job screen?
3. What does menopause mean to you? Do you think it is related to cardiovascular disease?
4. Do you think women prefer alternative/traditional medicine over western medicine for prevention of heart disease and managing menopause? Why?
5. Were the health coaching sessions, health monitoring and reminders useful?
6. Was tracking your blood pressure/weight/sugars (pick relevant parameter) convenient
7. How was your overall experience in this clinic? Were you satisfied with the services? (e.g. schedule, monitoring, touchpoints, accessibility to health care team)?
8. What do you think can be improved on?
9. What did you like best about it ?
10. Would you recommend other women to come and get screened or optimized for their overall CV health at mid-life?

**Semi-structured interview guide for healthcare providers**

***General questions***

1. Job title and role.
2. What are your thoughts on the women’s heart health preventive programme? Concerns? Benefits?

***Knowledge***

1. Why do you think women visit the doctor?
   Probe: Is it mainly for acute illnesses or job screen?
2. What is your understanding of the women’s heart health clinic service?
   Probe: What kind of conditions/What benefits
3. In your opinion, what do you think are the best ways to influence behaviour change in women have come forth for CV health check or have been diagnosed with CV condition?
4. Probe: Are there particular approaches that you think are or might be useful?

***Healthcare providers’ beliefs/perceptions, barriers and facilitators***

1. Do you think women prefer alternative/traditional medicine over western medicine for prevention of heart disease and managing menopause? Why?
2. Do you think the concept of preventive care is understood locally?
3. How many patients did you interact with? Could you describe your interaction with the patients? How long? What was satisfactory? What was not?
4. How is the experience of the programme different from standard care? What do you prefer?
5. What do you feel about participant adherence to the programme recommendations and follow-ups?
6. What do you think are possible barriers to implementing this program for stakeholder and patients point of view?
   -Probe: What are some of the reasons that you think will prevent women form seeing a doctor ? (eg. Too busy, housework, Costs)
7. What do you think are possible facilitators to this programme for stakeholder and patients' point of view?
   -Probe: Based on the COM-B model, eg. Knowledge, geography, resources, etc.)
8. Did you have enough support to implement the programme?
   Probe: Are the necessary resources available to the physician/nurses/health coach/case manager? Are there competing tasks or time constraints that affect whether patients get optimal care from the clinic?
9. What are your thoughts about implementing this intervention in regular care practice?
10. What do you think about the long-term sustainability of the intervention?

***Acceptance***

1. Do you think preventive heart health care should be provided for all women? (similar to mammogram/cervical cancer screening). Why?
2. What did you appreciate about the programme? What do you think are the benefits of the program?
3. Which aspects of the programme do you think are most/more useful?
4. Were there any parts you disliked? What was difficult about the implementing the programme? What could have been better?
5. How do you feel about the workflow in this programme?
   -Probes: Logistically (e.g. schedule, monitoring, touchpoints)? Experience? Is this better? In what way? Why? Why not?
6. From your experience, could you share a good experience during the implementation?
7. Could you share an experience that did not go as planned?
8. What are your hopes/changes/additions for the future of this programme in clinical practice?
9. What are your fears regarding the future use of this intervention in clinical practice?
10. Do you think this programme will improve women’s health and experience during the menopause transition in the short term?
11. Do you think this programme will, in the long-term result in positive health behavior changes and reduce cardiovascular disease burden in Singapore?
