# Supplementary material for: Evaluating a Preventive Heart Health Program for Women at Midlife: Protocol for a Mixed Methods Pilot Study
Source: JMIR Res Protoc. 2026 May 25;15:e83574. doi: 10.2196/83574 (PMC13200805; doi:10.2196/83574)
Supplement: Multimedia Appendix 4 [file resprot-v15-e83574-s004.docx]

| **Rating**  **No.** | **1** | **2** | **3** | **4** | **5** |
| --- | --- | --- | --- | --- | --- |
| **1. How satisfied are you with**  **the programme?** | Extremely satisfied | Satisfied | Neither satisfied nor dissatisfied | Dissatisfied | Extremely dissatisfied |
| **2. How satisfied are you with your interactions**  **with your doctor?** | Extremely satisfied | Satisfied | Neither satisfied nor dissatisfied | Dissatisfied | Extremely dissatisfied |
| **3. How satisfied are you with your interactions with your**  **health coach?** | Extremely satisfied | Satisfied | Neither satisfied nor dissatisfied | Dissatisfied | Extremely dissatisfied |
| **4. How easy was it for you to follow the recommenda tions for**  **monitoring your health?** | Extremely easy | Easy | Neither easy nor difficult | Difficult | Extremely difficult |
| **5. Were the goals and recommenda tions for diet and exercise convenient and**  **achievable?** | Extremely convenient | Convenient | Neither convenient nor inconvenien t | Inconvenien t | Extremely inconvenien t |
| **6. How likely will you continue to adhere to the health monitoring**  **after the programme?** | Extremely likely | Likely | Neither likely nor unlikely | Unlikely | Extremely unlikely |
| **7. How likely will you continue to adhere to diet and lifestyle recommenda tions after**  **the programme?** | Extremely likely | Likely | Neither likely nor unlikely | Unlikely | Extremely unlikely |

| **8. Do you think the programme interventions will help improve your health/help**  **you to age well?** | Extremely useful | Useful | Neither useful nor useless | Useless | Extremely useless |
| --- | --- | --- | --- | --- | --- |
| **9. How confident are you in achieving your health goals after**  **menopause?** | Extremely confident | Confident | Neither confident nor not confident | Not confident | Extremely not confident |
