# Supplementary material for: Evaluating a Preventive Heart Health Program for Women at Midlife: Protocol for a Mixed Methods Pilot Study
Source: JMIR Res Protoc. 2026 May 25;15:e83574. doi: 10.2196/83574 (PMC13200805; doi:10.2196/83574)
Supplement: Checklist 1 [file resprot-v15-e83574-s005.pdf]

The TIDieR (Template for Intervention Description and Replication) Checklist*:

Information to include when describing an intervention and the location of the information

Item number	Item	Where located **	
		Primary paper (page or appendix number)	Other † (details)
1.	**BRIEF NAME**<br>Provide the name or a phrase that describes the intervention.<br><br>An A-B-A reversal design [baseline (A), intervention (B), return to baseline (A)] will be used to assess the feasibility and acceptability of behavioural change strategy to increase heart-healthy habits in midlife women.	14	
2.	**WHY**<br>Describe any rationale, theory, or goal of the elements essential to the intervention.<br><br>There is a need for programmes of behavioural modification to be trialled in a controlled fashion and assessed rigorously for efficacy. Employing health services research and implementation science will be crucial in driving this strategy. Promising strategies on behaviour modification can be tested using multicentre implementation trials. The implementation of this programme in Singapore will have wider implications due to the ethnic differences in disease burden. Contextualising the customary practices and lifestyle factors including diet, exercise, smoking and psychosocial stress are important. Cultural attitudes to treatment adherence may also be	7	

	important and necessitates the health outcomes of patients.		
	**WHAT**		
**3.**	Materials: Describe any physical or informational materials used in the intervention, including those provided to participants or used in intervention delivery or in training of intervention providers. Provide information on where the materials can be accessed (e.g. online appendix, URL).<br><br>Information provided to the participants by the physicians and health coaches are aligned to the national clinical practice, dietary and physical activity guidelines, personalised where appropriate.	13-14	
**4.**	Procedures: Describe each of the procedures, activities, and/or processes used in the intervention, including any enabling or support activities.<br><br>Key features of the intervention include individualised risk assessment and sex-specific tailored care for women undergoing menopause transition. Women will have access to trained onsite personalised health coaching according to their CVD risk levels, health goals, and lifestyle. Collaborative consultations between the physician and health coach will include motivational interviewing to identify any barriers causing the knowledge and execution gap, to target change in both internal and external factors of behavioural determinants, ultimately attaining health goals. Women are encouraged to track their health parameters using the existing hospital e-health platform between the follow-ups. Timely encouragements and reminders that are relevant to individuals' target that was set together with the health coach will also be sent via the electronic health platform.	13-14	

	**WHO PROVIDED**		
**5.**	For each category of intervention provider (e.g. psychologist, nursing assistant), describe their expertise, background and any specific training given.<br><br>Health coaching sessions will be conducted by certified health coaches within the hospital sites on a one-to-one basis in the Women's Heart Health clinic.	13	
	**HOW**		
**6.**	Describe the modes of delivery (e.g. face-to-face or by some other mechanism, such as internet or telephone) of the intervention and whether it was provided individually or in a group.<br><br>Health coaching sessions will be conducted by certified health coaches within the hospital sites on a one-to-one basis in the Women's Heart Health clinic.	13	
	**WHERE**		
**7.**	Describe the type(s) of location(s) where the intervention occurred, including any necessary infrastructure or relevant features.<br><br>Health coaching sessions will be conducted by certified health coaches within the hospital sites on a one-to-one basis in the Women's Heart Health clinic.	13	
	**WHEN and HOW MUCH**		
**8.**	Describe the number of times the intervention was delivered and over what period of time including the number of sessions, their schedule, and their duration, intensity or dose.<br><br>Bi-weekly health coaching will be introduced over a 6-month intervention period.	14	
	**TAILORING**		

**9.**	If the intervention was planned to be personalised, titrated or adapted, then describe what, why, when, and how.<br><br>Collaborative consultations between the physician and health coach will include motivational interviewing to identify any barriers causing the knowledge and execution gap, to target change in both internal and external factors of behavioural determinants, ultimately attaining health goals.	13<br>_	
	**MODIFICATIONS**		
**10.‡**	If the intervention was modified during the course of the study, describe the changes (what, why, when, and how).	N/A<br>_	
	**HOW WELL**		
**11.**	Planned: If intervention adherence or fidelity was assessed, describe how and by whom, and if any strategies were used to maintain or improve fidelity, describe them.<br><br>We used Proctor's implementation conceptual framework and the overarching RE-AIM framework to assess the intervention fidelity using the following strategies:<br>• **Reach**: number, proportion, and representativeness of the women participating in the programme.<br>• **Effectiveness**: degree to which participants engage in healthy heart behaviours and the degree of change in clinical CVD indicators and QOL measures. Both behavioural change and patient-related outcome indicators are measured.<br>• **Adoption**: programme strategies by the healthcare providers in the Women's Heart Health Clinic will be reviewed, from the perspective of physicians, allied health	10-13<br>_	

	professionals, and the clinical operations team.<br>• **Implementation**: evaluation and feedback, training and supporting stakeholders, adaptation and tailoring, infrastructure change, and financial strategies.<br>• **Maintenance:** feasibility, sustainability, service outcomes, and participant satisfaction in forms of observations, audits and feedbacks.		
**12.‡**	Actual: If intervention adherence or fidelity was assessed, describe the extent to which the intervention was delivered as planned.	N/A<br>______________<br>_	______________

** **Authors** - use N/A if an item is not applicable for the intervention being described. **Reviewers** – use '?' if information about the element is not reported/not sufficiently reported.

† If the information is not provided in the primary paper, give details of where this information is available. This may include locations such as a published protocol or other published papers (provide citation details) or a website (provide the URL).
‡ If completing the TIDieR checklist for a protocol, these items are not relevant to the protocol and cannot be described until the study is complete.

* We strongly recommend using this checklist in conjunction with the TIDieR guide (see *BMJ* 2014;348:g1687) which contains an explanation and elaboration for each item.

* The focus of TIDieR is on reporting details of the intervention elements (and where relevant, comparison elements) of a study. Other elements and methodological features of studies are covered by other reporting statements and checklists and have not been duplicated as part of the TIDieR checklist. When a **randomised trial** is being reported, the TIDieR checklist should be used in conjunction with the CONSORT statement (see www.consort-statement.org) as an extension of **Item 5 of the CONSORT 2010 Statement.** When a **clinical trial protocol** is being reported, the TIDieR checklist should be used in conjunction with the SPIRIT statement as an extension of **Item 11 of the SPIRIT 2013 Statement** (see www.spirit-statement.org). For alternate study designs, TIDieR can be used in conjunction with the appropriate checklist for that study design (see www.equator-network.org).
